# Case Report: Electro-cortical network effects of an acute stroke revealed by high-density electroencephalography

**DOI:** 10.3389/fnins.2025.1486249

**Published:** 2025-05-15

**Authors:** Elena Monai, Brinda Sevak, Klevest Gjini, Lorenzo Pini, Giulio Tononi, Aaron F. Struck, Melanie Boly

**Affiliations:** ^1^Department of Neurology, University of Wisconsin–Madison, Madison, WI, United States; ^2^Wisconsin Institute for Sleep and Consciousness, University of Wisconsin–Madison, Madison, WI, United States; ^3^Padova Neuroscience Center, University of Padua, Padua, Italy; ^4^William S. Middleton Veterans Administration Hospital, Madison, WI, United States

**Keywords:** HD-EEG, stroke, network, diaschisis, slow wave

## Abstract

**Introduction:**

Focal lesions such as a stroke can cause not only local effects but also distant effects in anatomically intact regions. The impact of stroke lesions on brain networks has been mapped using neuroimaging techniques such as functional magnetic resonance imaging (fMRI) and diffusion tensor imaging (DTI). In the present study, we established the feasibility of detecting network dysfunction at the electrocortical level using high-density electroencephalography (HD-EEG).

**Case report:**

We studied brain function using HD-EEG in a patient with an acute left middle cerebral artery stroke. Slowing in the delta range was present beyond the ischemic focus, extending to the perilesional regions as well as distant regions. There was also delta connectivity in the stroke hemisphere with slowing affecting mainly distant regions that were interconnected with the site of the ischemic region (i.e., network-level diaschisis), although this was not statistically significant.

**Conclusion:**

This case study illustrates the feasibility of using HD-EEG to map local and distant electrical activity consequences of an acute stroke on cortical functions. Such a technique could be clinically useful to improve personalized stroke-network mapping in patients with acute cortical lesions.

## Introduction

Stroke affects nearly 800,000 individuals only in the United States each year and is one of the main causes of disability in adults (Dobkin, 2005). In contrast with the significant progress in acute therapy (i.e., thrombolysis and thrombectomy), there have not been rehabilitation therapeutic breakthroughs enabling patients to substantially recover from neurological deficits beyond 24 h after stroke (Grefkes and Fink, 2020). New strategies to improve recovery after stroke may benefit from a better understanding of network effects induced by focal brain lesions. Indeed, it is currently well recognized that focal lesions, such as stroke, cause direct and indirect effects that extend beyond the borders of the primary anatomical lesions (Carrera and Tononi, 2014).

Here, the concept of diaschisis refers to any remote alteration directly caused by the lesion, including a spectrum of neurophysiological effects, from focal to connectomal (Carrera and Tononi, 2014).

The underlying network alterations have been studied mainly via neuroimaging techniques such as functional magnetic resonance imaging (fMRI), diffusion tensor imaging (DTI), and positron emission tomography (PET) (He et al., 2007; Baldassarre et al., 2016; Forkel and de Schotten, 2020). An important result derived from this new network-based perspective has been the evidence that functional disconnection correlates with neurological deficits and behavioral outcomes after stroke and the normalization of the functional connectivity patterns predicts longitudinal behavioral recovery (Siegel et al., 2016). This evidence is relevant for its clinical implications, such as the adoption of neuromodulation aiming at restoring network dysfunction (Griffis et al., 2020).

Network effects of focal lesions can be of multiple natures (e.g., functional, structural, and electro-cortical). Despite the extensive adoption of fMRI and DTI in research studies, these techniques do not allow us to distinguish areas with slow-wave activities (similar to sleep and thus potentially “arousable”) that could be potential targets of neuromodulation versus silent (and therefore irreversibly damaged) areas. Moreover, the high cost of fMRI and DTI neuroimaging techniques limits their application in clinical practice.

Transcranial magnetic stimulation–EEG (TMS-EEG) is a promising tool that can distinguish between inactivated regions versus regions with slow-wave activities (Sarasso et al., 2020)_._ However, this technique requires prolonged acquisitions. High-density EEG (HD-EEG) also can distinguish between regions that are silent versus regions displaying slow activity, and it is more portable and lower cost. HD-EEG overcomes the limit of the low spatial resolution of standard EEG. HD-EEG quantitative analysis has been demonstrated to be more sensitive to detecting focal slowing (Boly et al., 2017) and allows us to perform connectivity analysis in source space that is more comparable to previous fMRI connectivity studies. So far, only a minority of studies have applied HD-EEG in acute stroke patients. The studies show the presence of slow-wave activity in the delta and theta range at the scalp level ipsilateral to the infarct (Poryazova et al., 2015). Some studies also observe an increase in delta power in the contralateral hemisphere (Assenza et al., 2013) suggesting distant effects.

However, compared to these previous studies, we wanted to highlight the feasibility and the significance of mapping the local and network consequences of focal lesions using HD-EEG in the acute phase as the starting point for clinical/therapeutic applications that, of necessity, need to be personalized and detailed.

In other words, the mapping of the unique neurophysiological signatures of the patient and his/her neurological deficits is the conditio sine qua non if we want to implement neuromodulation strategies in the clinical practice at the bedside for a particular patient.

Moreover, this single study’s HD-EEG source reconstruction was not applied in previous studies to distinguish more accurately the slowing in lesional vs. perilesional or remote areas. First, we quantified the presence and the cortical source of local and distant slowing associated with the ischemic/hypoperfused regions. Second, an explorative connectivity analysis was performed to search for possible long-range connectivity effects in the delta band induced by the focal lesion.

## Materials and methods

### Case description

**Figure 1:**
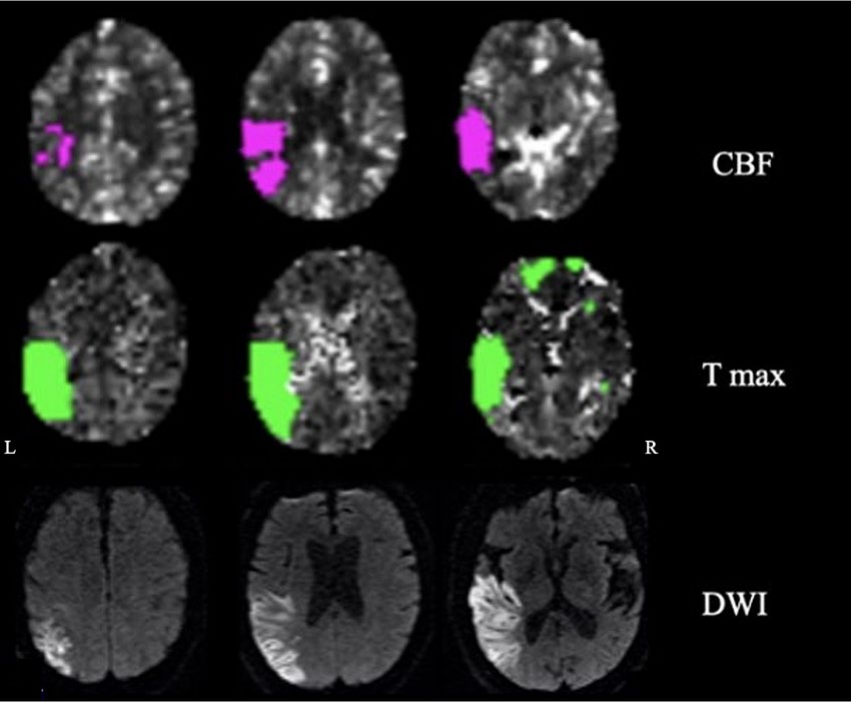
Neuroimaging: cerebral blood flow (CBF), time-to-maximum (Tmax), and diffusion-weighted imaging (DWI) showing penumbra and ischemic regions.

The patient was a 72-year-old man with a history of atrial fibrillation and abdominal aortic aneurysm repair presenting with a wake-up stroke with right-sided weakness and facial drop, and aphasia. CT with perfusion and angio-CT at 3.5 h from onset showed infarct in the left middle cerebral artery (MCA) territory (mismatch volume of 62 mL and ratio of 2.3) with a left MCA thrombus. Neuroimaging was reviewed by two neurologists; the ischemic core involved the following regions: superior and middle temporal gyrus (excluding the pole), inferior parietal lobule and supramarginal gyrus, posterior insula, and entorhinal cortex. The penumbra region extended to the inferior temporal gyrus including the pole, superior parietal lobe, and parahippocampal (Figure 1).

The patient underwent thrombectomy and arterial tissue plasminogen activator (tPA). On day 3, at the neurological examination, there were mild weakness of the right upper extremity, expressive and receptive aphasia, spatio-temporal disorientation, and right-side hemispatial and body neglect. The MRI on day 4 confirmed residual ischemic damage of the left inferior parietal lobule, temporal lobe (excluding the pole), and posterior insula (Figure 1).

Following the acute phase, the patient was transferred to the rehabilitation unit and subsequently underwent angioplasty and stenting of the left internal carotid artery. At 1-year follow-up visit, the patient reported having mild aphasia and right-side neglect.

### HD-EEG acquisition

Resting-state awake HD-EEG was performed on the same day of the MRI (day 4). Data were recorded using 128-channel dense-array electrodes EEG sensor net (Compumedics) at a sampling rate of 1,000 Hz. Electrode impedances were set below 50 kΩ. The recording lasted for 83 min.

### Data preprocessing

EEG data were analyzed in MATLAB using custom scripts and the EEGLAB toolbox (Delorme and Makeig, 2004). First, the raw data were sleep-scored using the standard AASM (American Academy of Sleep Medicine) sleep scoring guidelines (Berry et al., 2015) to confirm the presence of only wake data in the files. EEG signals were then bandpass filtered between 0.5 and 25 Hz using a hamming window. Subsequently, bad channels and bad stretches were visually identified and rejected. Fast Fourier Transform (FFT)-based power was then calculated, and the topographical maps of delta, theta, and alpha frequency bands were used to further detect and remove any remaining bad channels. Independent component analysis using the AMICA (Palmer et al., 2011) toolbox was applied to remove artifactual components such as eye blinks, muscle artifacts, heartbeats, and channel noise. Multiple iterations of AMICA were used to reject segments where decomposition failed, so optimizing decomposition before rejecting artifact components in the signal. The signal was then average referenced after interpolating the bad channels.

### Power spectrum analysis

FFT analysis was performed on the cleaned data using 6 s of non-overlapping windows to calculate the Welch’s power in delta (1–4 Hz), theta (4–8 Hz), alpha (8–12 Hz), and beta (12–25 Hz) frequency bands. Topographical maps were plotted using 128 channels. Absolute powers were calculated for each of the delta–alpha frequency band.

Delta-to-alpha ratio (DAR) and delta–theta to alpha–beta ratio (DTABR) were also calculated within each hemisphere, to estimate inter-hemispheric asymmetries.

For the control subject (M, 60 years old), the original data acquired using 256-channel dense-array electrodes EEG net (EGI) were first transformed to 128-channel Compumedics array format, and then, the same analysis of wake epochs described above for the stroke patient was performed.

### Delta source reconstruction and connectivity analysis

Cortical surface source localization was performed on the HD-EEG data from the patient and the control using Brainstorm (Tadel et al., 2011). Using MNI/ICBM152 (Fonov et al., 2011) default anatomy, a head model was created for each subject using forward modeling with OpenMEEG BEM (Gramfort et al., 2010). Scalp data were then projected to source space using the current density map (dSPM) (Dale et al., 2000) method of minimum norm imaging for unconstrained dipole orientations. Source space power spectral density was calculated using Welch’s power estimate method for the delta frequency band.

The anatomical localization of delta power after source reconstruction was reviewed by two neurologists. Mindboggle-101 (Klein and Tourville, 2012) atlas from the Mindboggle project with 62 regions of interest (ROI) was used to define ROIs for connectivity analysis. Lagged coherence (Pascual-Marqui, 2007) connectivity measure was used to overcome the artifacts caused due to volume conduction and spatial leakage. The connectivity matrix for the delta frequency band was computed for brain network analysis using graph theory principles. The adjacency/connectivity matrix was thresholded to keep only the strongest 50% of the connections using lagged coherence. Brain connectivity toolbox (Rubinov and Sporns, 2010) in MATLAB was used to study the complex brain networks from the lagged coherence connectivity matrix.

Specifically, the following graph measures were computed: degree (i.e., number of connections of a particular brain region with the rest of the regions), strength (analogous to degree and symbolizes the strength of neural communication between brain regions), eigenvector centrality (influence of a brain region in the network), local efficiency (how the information is exchanged within the neighborhood of the brain region thus measuring the robustness of the network), and clustering coefficient (to study the integration of the brain networks).

### Statistical analysis

Statistical analysis was performed to compare absolute power for each frequency band within the patient (i.e., lesional vs. contralesional hemisphere) and between the patient and the control (between homologous hemispheres) using an independent-sample *t*-test between the mean values within each hemisphere. DAR and DTABR were also calculated and compared between hemispheres in the stroke patient using a two-sample *t*-test (*p* < 0.05).

## Results

### HD-EEG power spectrum

**Figure 2:**
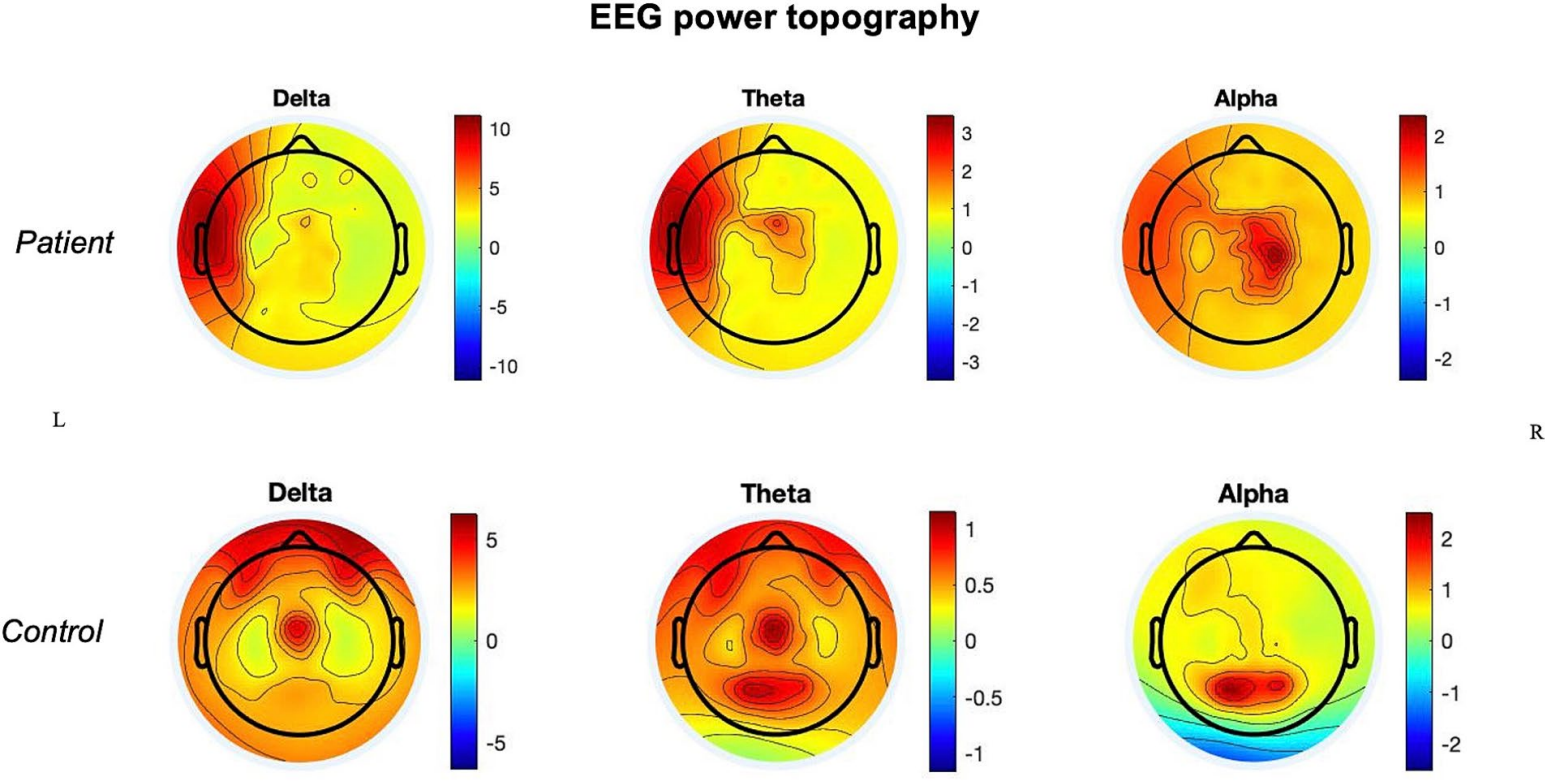
Power topography: power topographical analysis of delta, theta, and alpha frequency bands in the stroke patient and in the control.

**Figure 3:**
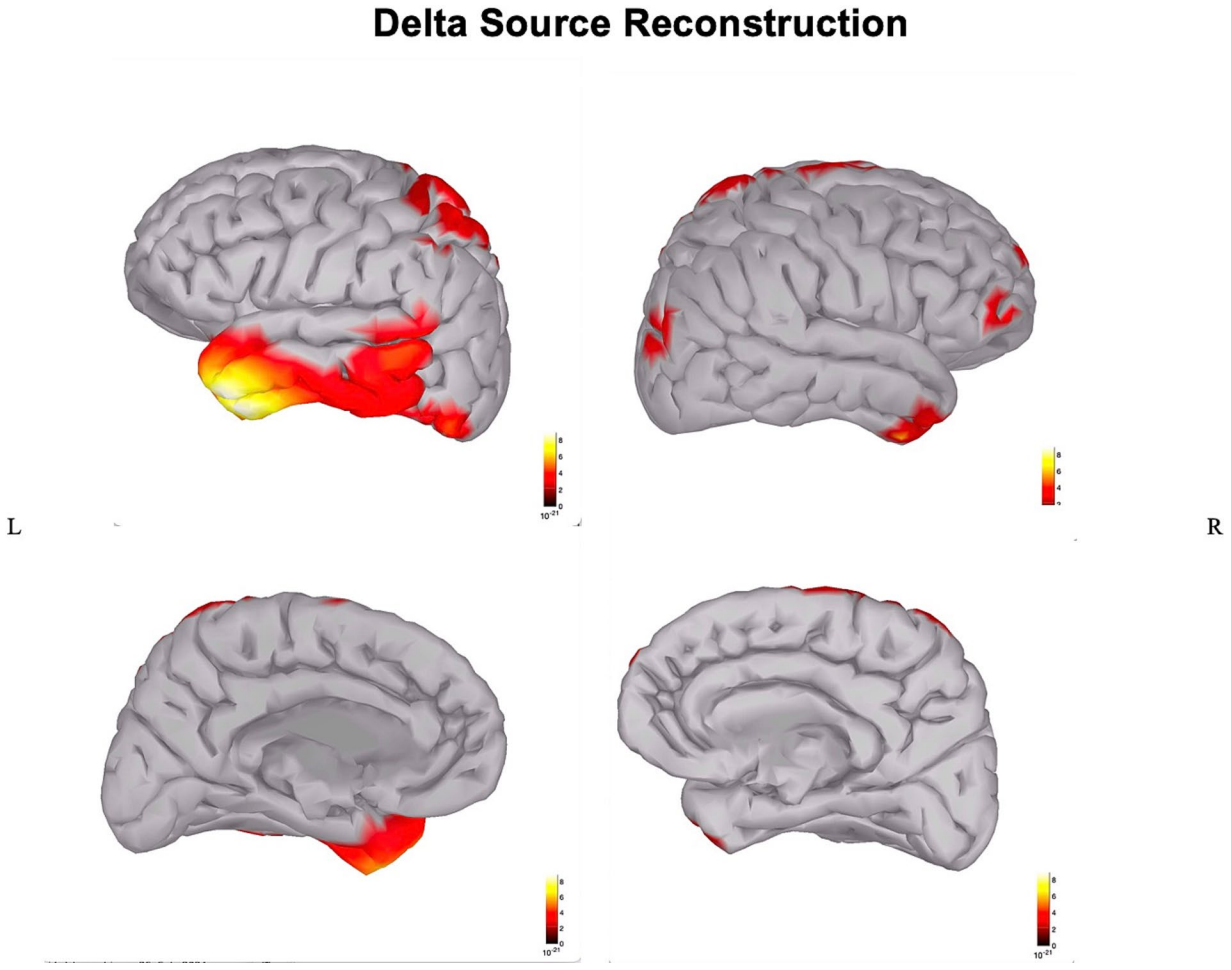
Source reconstruction of delta power: source reconstruction of delta power in the stroke patient.

In the patient, the ischemic lesion involved the left temporal lobe (but not the temporal pole) and the adjacent inferior parietal lobule (Figure 1). The power spectrum analysis of HD-EEG revealed a significant asymmetry between the two hemispheres with predominant delta and theta activity (*p* < 0.05) over the lesioned hemisphere. Both scalp topographical analysis and source reconstruction provided evidence for an increase in delta activity bilaterally in ventral regions (including the temporal poles) and the superior parietal lobules and in the left post-central gyrus (Figures 2, 3).

In comparison with the control, there were higher absolute values of delta and theta power in the lesioned hemisphere and of theta power in the non-lesioned hemisphere.

Qualitatively, at the topographical level, both hemispheres of the patient also showed the absence of alpha activity over the occipital regions, which were not part of the ischemic core, with an alpha posterior-to-anterior shift.

In the patient, the values of DAR and DTABR were overall significantly higher (p < 0.05) in the lesioned hemisphere than in the contralateral hemisphere.

### HD-EEG connectivity

**Figure 4:**
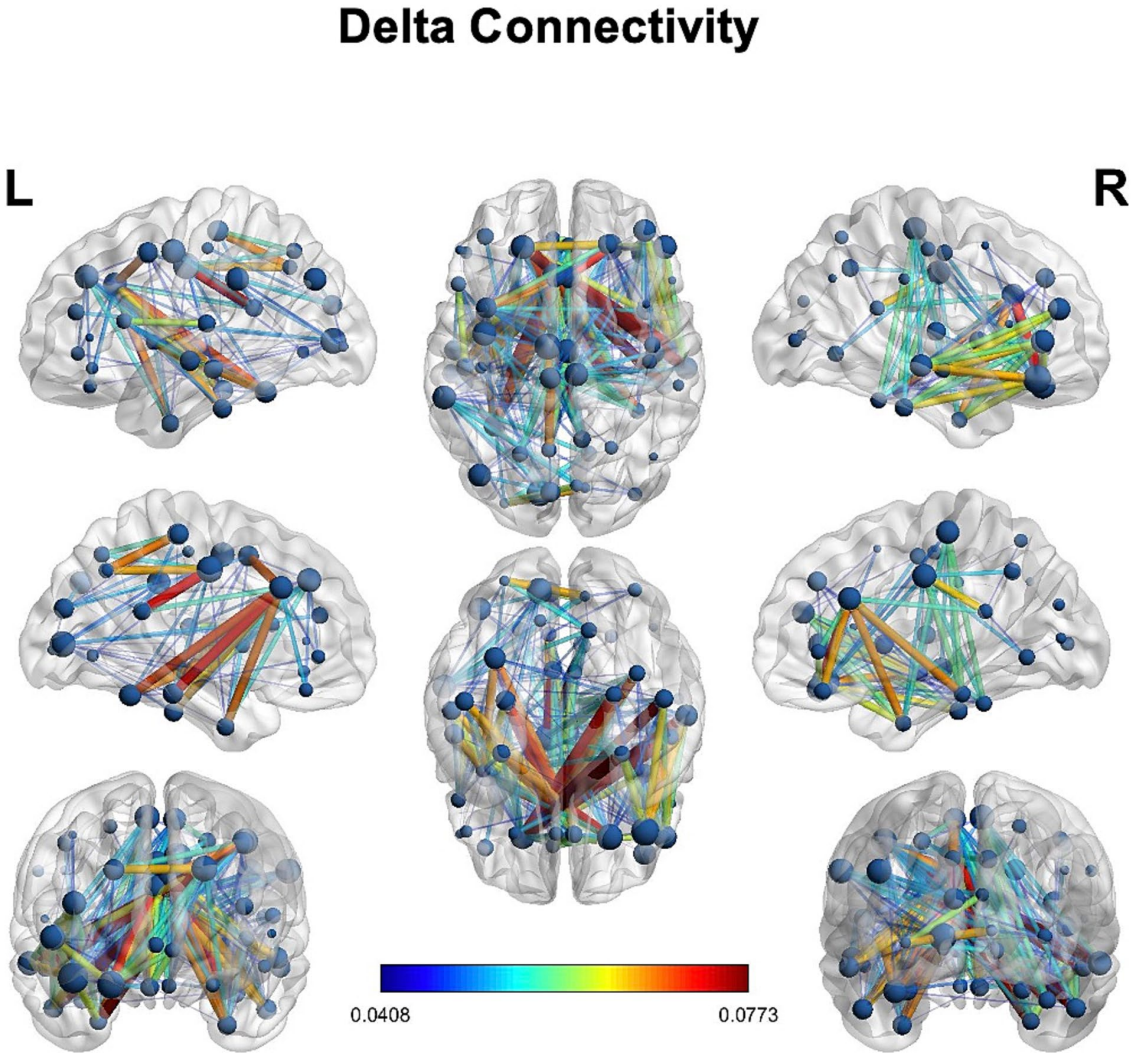
Delta connectivity: connectivity in the delta power in the stroke patient.

We observed delta connectivity (degree, strength, eigenvector centrality, local efficiency, and clustering coefficient) in the lesioned hemisphere with the rest of the brain (Figure 4). However, none of the differences was statistically significant at *p* < 0.05.

## Discussion

The main findings of this HD-EEG stroke case study were (1) the presence of delta activity beyond the ischemic focus, in both perilesional and distant regions (Figures 2, 3); (2) the loss of posterior alpha activity (Figure 2); and (3) delta connectivity over the stroke hemisphere (Figure 4).

Our findings of percolation of slow-wave activity around the ischemic core are in line with studies using TMS-EEG, which revealed the presence of sleep-like responses during wake over the perilesional areas (Sarasso et al., 2020). Considering that HD-EEG, as shown in this study, has the advantages of being more feasible and accessible than TMS-EEG, its application is promising on a larger scale for stroke patients that could benefit from neurophysiological brain mapping.

Similarly, in recent years, studies applying EEG and HD-EEG in acute stroke patients have found distant neurophysiological effects ipsilateral to the infarct (Poryazova et al., 2015), but with also some evidence in the contralateral hemisphere (Assenza et al., 2013; Wu et al., 2016).

However, only a minority of the studies applied HD-EEG, despite the fact that HD-EEG provides better quality of the signal and better data for more sophisticated and detailed analysis (Lanzone et al., 2023), and it is more sensitive in the detection of focal slowing (Boly et al., 2017).

Here, we applied HD-EEG to obtain an individualized neurophysiological mapping and, implementing the use of source reconstruction analysis, we could clarify how slowing was localized not only in the lesion but also in specific perilesional and contralateral regions that are interconnected with the infarct area (Figure 3).

A post-stroke individual and high-spatial resolution mapping approach can ultimately better translate into the development of neuromodulatory strategies for rehabilitation that, of necessity, need to be personalized.

Of note, in our patient, there was also a loss of alpha power over the occipital regions (Figure 2). As these regions were neither ischemic nor hypoperfused, this effect may also represent a sign of long-range cortical–subcortical effect via the thalamus and inter-hemispheric (dis)-connections (Juhász et al., 1997).

Understanding network effects induced by focal lesions has relevant clinical implications. Neurological symptoms, especially cognitive deficits, seem to correlate better with their impact on brain networks, rather than solely the lesion site (Siegel et al., 2016). This has already been demonstrated in syndromes like neglect using fMRI and DTI [e.g., functional impairment of the dorsal attention network (DAN) induced by lesions belonging to the ventral attention network (VAN) (Corbetta and Shulman, 2011); increased latencies of visual evoked potentials in neglect patients (Spinelli et al., 1994)].

Interestingly, our patient had right-sided neglect fitting with evidence on HD-EEG of delta activity over the left superior parietal lobule (i.e., the posterior node of the left dorsal attention network) vis-a-vis a lesion involving the ventral system (Figure 3).

Instead, there was a difference in the involvement of the two language streams (ventral affected vs. dorsal not affected).

Intriguingly, at the neurological follow-up in the chronic phase, neglect was still present while aphasia was improved.

We hypothesized that the different degrees of involvement of the 2 function-related systems could account for the different evolution of the deficits in the recovery phase.

Understanding the cortical topography of the network dysfunction via HD-EEG could be predictive of behavioral deficits in a way complementary to fMRI and DTI. However, detailed neuropsychological assessment in the acute and chronic phases is necessary. Moreover, an exploratory connectivity analysis on delta power using graph theory showed the presence of delta connectivity in the lesioned hemisphere (Figure 4), though without reaching statistical significance in the difference with the contralateral hemisphere. Future studies may try to investigate whether delta connectivity” reflects a pathological synchronization of cortical regions (due to disconnection) or a reflection of plastic changes in areas interconnected with the focal lesion.

## Conclusion

In this case report, we confirmed the feasibility of performing HD-EEG in the acute phase of stroke to quantify the network effects of focal lesions. HD-EEG analysis revealed the presence of slowing not only around the lesion but also in specific cortical regions distant from the stroke, prominently in the ipsilateral hemisphere (Figures 2–4).

For this case study, the comparison between neuroimaging and delta source maps was based on the review of the two modalities by clinical experts.

For future studies, multimodal analysis would benefit from the development/use of software/toolboxes able to easily represent different signals on the patient’s anatomical brain. Studies with cohorts of stroke patients and controls may further clarify the relationship between electro-cortical disconnection observed with HD-EEG and the structural and functional disconnections induced by focal lesions quantified by fMRI, DTI, and PET (Monai et al., 2023). Finally, the correlation between electro-cortical network effects and clinical deficits needs to be further investigated in future studies.

In the long run, electro-cortical network disconnection with HD-EEG may become a personalized predictive biomarker to guide rehabilitation strategies with neuromodulation techniques. In particular, neuromodulation strategies may aim to target distant regions that are dysfunctional (and therefore likely contributing to the neurological deficits), though still anatomically intact, and therefore potentially “arousable,” with the final aim to improve the recovery from neurological/neuropsychological deficits. In our patient, we would expect that neuromodulation of the dorsal attention network (shown to be deactivated by HD-EEG while still intact Figure 3), could contribute to the recovery from neglect.

In conclusion, we show that modern tools of EEG, in particular the use of source reconstruction with HD-EEG to better increase spatial information, can gain insightful findings when applied to individual patients to envision a personalized neuromodulation approach.

## Data Availability

The datasets presented in this article are not readily available because of ethical and privacy restrictions. Requests to access the datasets should be directed to the corresponding author/s.
